# Pan-cancer analysis of T-cell proliferation regulatory genes as potential immunotherapeutic targets

**DOI:** 10.18632/aging.205977

**Published:** 2024-06-29

**Authors:** Ruqiong Wei, Shihui Xiao, Shijian Zhao, Wenliang Guo, Ying Liu, Marìa del Mar Requena Mullor, Raquel Alarcòn Rodrìguez, Qingjun Wei, Yinteng Wu

**Affiliations:** 1 Department of Rehabilitation Medicine, The First Affiliated Hospital of Guangxi Medical University, Nanning, Guangxi 530021, China; 2 Department of Orthopedic and Trauma Surgery, The First Affiliated Hospital of Guangxi Medical University, Nanning, Guangxi 530021, China; 3 Department of Cardiology, The Affiliated Cardiovascular Hospital of Kunming Medical University (Fuwai Yunnan Cardiovascular Hospital), Kunming, Yunnan 650000, China; 4 Department of Rehabilitation Medicine, The Eighth Affiliated Hospital of Guangxi Medical University, Guigang, Guangxi 537100, China; 5 Faculty of Health Sciences, University of Almerìa, Carretera de Sacramento, Almeria 04120, Spain

**Keywords:** T-cell proliferation regulatory genes, T cells, CD8+ T cells, CD4+ T cells, cancer, tumor immune microenvironment (TIM)

## Abstract

T cells are the key to killing tumor cells. However, the exact mechanism of their role in cancer is not fully understood. Therefore, a comprehensive understanding of the role of T-cell proliferation regulatory genes in tumors is needed. In our study, we investigated the expression levels of genes controlling T-cell proliferation, their impact on prognosis, and their genetic variations. Additionally, we explored their associations with TMB, MSI, ESTIMATEScore, ImmuneScore, StromalScore, and immune cell infiltration. We examined the role of these genes in cancer-related pathways using GSEA. Furthermore, we calculated their activity levels across various types of cancer. Drug analysis was also conducted targeting these genes. Single-cell analysis, LASSO Cox model construction, and prognosis analysis were performed. We observed distinct expression patterns of T-cell proliferation regulatory genes across different malignant tumors. Their abnormal expression may be caused by CNA and DNA methylation. In certain cancers, they also showed complex associations with TMB and MSI. Moreover, in many tumors, they exhibited significant positive correlations with ESTIMATEScores, ImmuneScore, and StromalScore. Additionally, in most tumors, their GSVA scores were significantly positively correlated with various T-cell subtypes. GSEA analysis revealed their involvement in multiple immune pathways. Furthermore, we found that model scores were associated with patient prognosis and related to tumor malignancy progression. T-cell proliferation regulatory genes are closely associated with the tumor immune microenvironment (TIM), especially T cells. Targeting them may be an essential approach for cancer immunotherapy.

## INTRODUCTION

Solid tumors are highly complex tissues that contain highly heterogeneous cancer cells and a tumor microenvironment (TME) composed of immune cells, stromal cells, blood vessels/lymphatics, nerve endings, and extracellular matrix (ECM). Among them, various signaling molecules act as immunomodulatory microenvironments to continuously reshape local immunity. The tumor immune microenvironment (TIME) is an immune system composed of different cell groups and their interactions in the TME ecological niche. It has been suggested to play a key role in cancer development, progression, and therapeutic response [1–3]. T cells of the immune system play a crucial role in identifying and eliminating cells that pose a threat to the body, such as infected and cancerous cells [4]. The ultimate goal of tumor immunotherapy is to eradicate cancer cells. CD8+ cytotoxic T lymphocytes (CTL) are vital immune surveillance cells and their abundant presence in tumor tissues serves as a positive prognostic indicator. Increasing the proportion of CTL with killing function in patients’ tumor tissues can help inhibit tumor progression or even achieve complete elimination [5, 6].

In patients with chronic infections and cancer, T cells undergo continuous stimulation due to prolonged exposure to persistent antigens and inflammation. This persistent stimulation leads to the exhaustion of T cells, resulting in the loss of their effector functions and the absence of memory T cell characteristics. This state is referred to as T cell exhaustion [7, 8]. CD8+ T cells are key mediators of cytotoxic effector function in infection, cancer, and autoimmunity. Persistent exposure to antigens and activating signals (e.g. chronic viral infections or cancer), or lack of support from CD4+ T cells and immune-supporting cytokines, among others, will lead to differentiation of CD8+ T cells into failing T cells [9]. This is a progressive differentiation process controlled by specific transcriptional mechanisms, gene expression profiles, metabolic alterations, and epigenetic background. In addition, immunomodulatory cells such as regulatory T cells, tumor-associated macrophages, and dendritic cells can regulate T cell immune response and promote T cell failure through the production of immunosuppressive metabolites and the depletion of immune support nutrients [10–14]. Coordination of these immunomodulatory cells and tumor cells in TME can impose metabolic stress on tumor-infiltrating lymphocytes (TIL), thereby eliminating the anti-tumor response of T cells. In most solid tumors, the presence of a high number of infiltrating CD8+ T cells is beneficial for tumor treatment [15–17]. However, in the case of RCC (renal cell carcinoma), a high infiltration of CD8+ T cells is associated with a poor prognosis [18]. Treg (regulatory T cells) are a subset of CD4+ T cells that have immunosuppressive properties and play a crucial role in maintaining self-tolerance and immune homeostasis. In the context of tumor immunity, Treg hinders the immune surveillance of cancer in healthy individuals and suppresses the anti-tumor immune responses of the host. Consequently, this leads to tumor progression in various types of cancer [19, 20]. Foxp3+ regulatory T cells (Treg) promote tumor immune escape by forming a suppressive tumor microenvironment. Therefore, strategies targeting Treg may help to enhance the efficacy of immune checkpoint blockade (ICB) against cancer [21]. There are significant molecular differences between γδ T cells in the normal intestinal epithelium and γδ T cells in CRC tumors, and they play opposite roles in CRC progression - γδ T cells in normal epithelial tissue play an anti-tumor role, while γδ T cells enriched in tumors mostly “defect” as cancer pushers, and this contrasting cellular function is associated with changes in the T cell receptors used [22]. Further research is needed to explore the functional role of T cells in the tumor microenvironment and their underlying mechanisms, particularly in different cancer types.

Thanks to advances in bioinformatics, researchers have recently screened a variety of regulatory genes that promote or inhibit T cell function through the overexpression of a large-scale genome-wide open reading frame library, which has recently been published in Nature. These genes increase the proliferation of human CD4+ and CD8+ T cells and activate the secretion of key cytokines, providing new strategies to optimize and improve T cell therapy. In this study, we conducted a systematic analysis of T cell proliferation regulators and their impact on the prognosis of cancer patients. Additionally, we investigated the genomic and epigenetic alterations associated with these regulators. We also investigated their relationship with the immune microenvironment, cancer-related pathways, and especially immune-related pathways. We also investigated the drugs that can act on them. Finally, single-cell analysis, LASSO Cox model construction and prognostic analysis were performed.

## RESULTS

### Expression and survival analysis results

**Figure 1:**
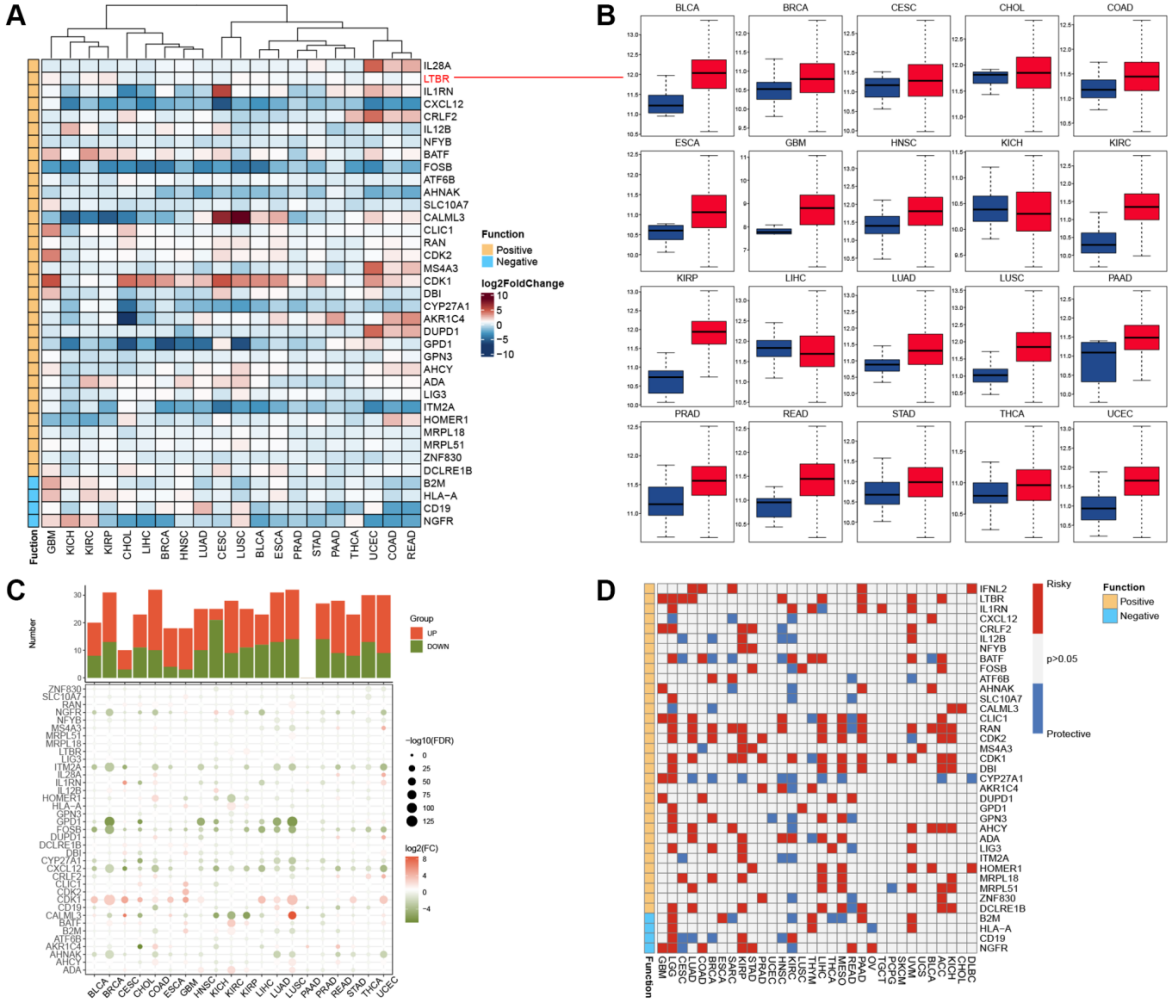
**Expression and prognostic analysis.** (**A**) The gene expression of T cell proliferation regulatory genes in cancers. (**B**) Box plots showing the expression distribution of LTBR across tumor and normal samples. (**C**) Histogram (upper panel) shows the number of significantly differentially expressed genes, and the heatmap shows the fold change and FDR of T cell proliferation regulatory genes in each cancer. (**D**) Summary of the correlation between expression of T cell proliferation regulatory genes and patient survival.

We included twenty cancers with paired normal and tumor samples from the TCGA database for our analysis. Through differential analysis of the expression levels of T-cell proliferation-related genes, we observed that CALML3 exhibited the highest expression levels in LUSC and CESC (Figure 1A). Figure 1B shows that the most functional T-cell proliferation-related genes, LTBR, have low expression in KICH and LIHC and high in 18 other cancers. Using genes with FoldChange >1 and adjusted *P*-values less than 0.05 as truncation criteria, we found that T-cell proliferation-associated genes were mostly significantly high-expressed (Figure 1C). We also identified the relationship between T-cell proliferation-related genes and survival in cancer patients and found that they play a protective or risk factor (Figure 1D).

### Genetic analysis

**Figure 2:**
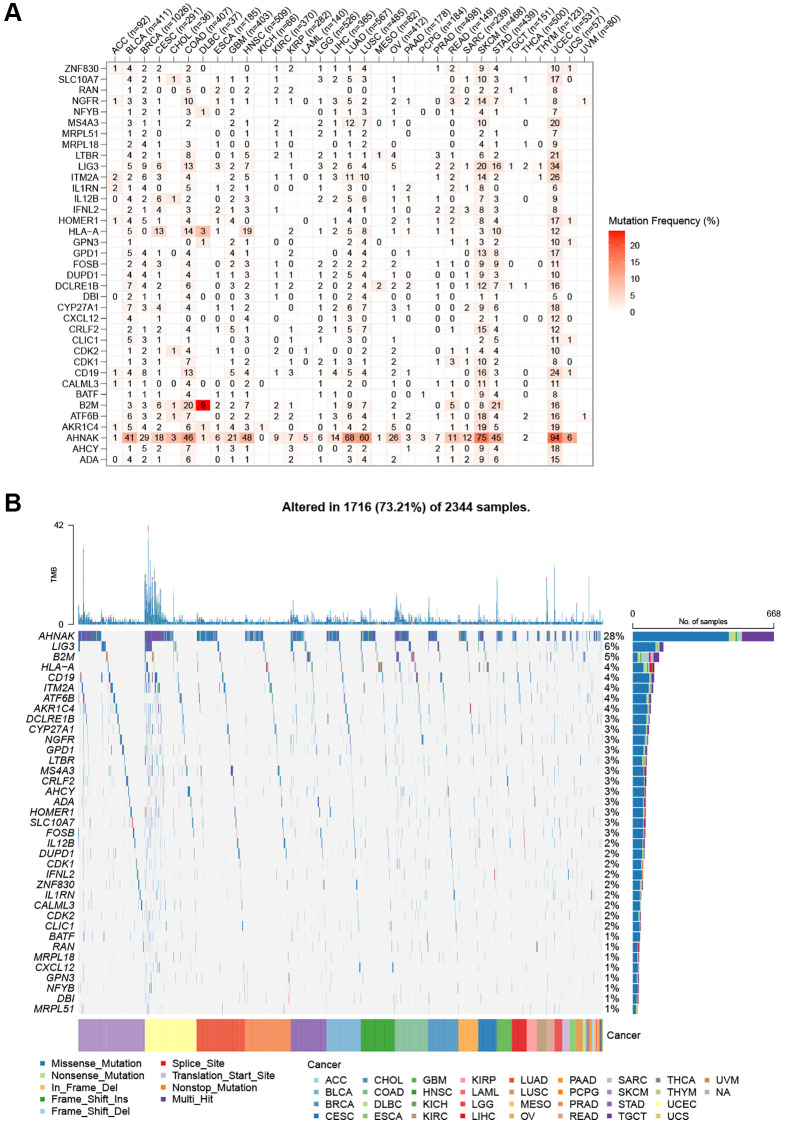
**SNV frequency and variant types of T cell proliferation regulatory genes.** (**A**) Mutation frequency of T cell proliferation regulatory genes. (**B**) SNV oncoplot. An oncoplot showing the mutation distribution of T cell proliferation regulatory genes and a classification of SNV types.

**Figure 3:**
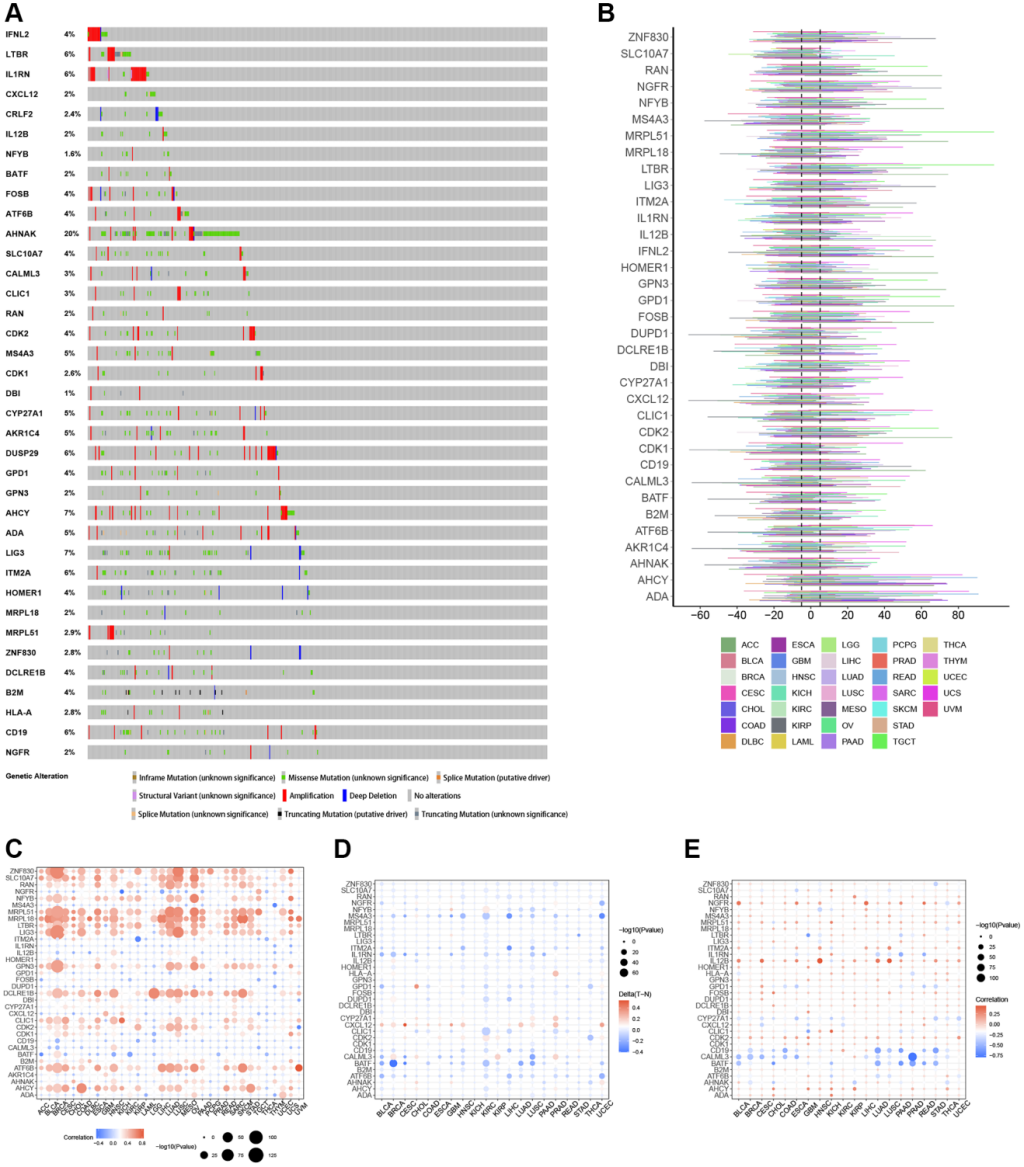
**Genomic and epigenetic alterations.** (**A**) Type of genetic variation. (**B**) The frequency of somatic copy number alterations. (**C**) The correlation between somatic copy number alterations and the expression of genes. (**D**) Differential methylation of genes in cancers. (**E**) The correlation of gene expression and promoter methylation.

By examining SNP data, we identified the frequency and type of mutations of T-cell proliferation-associated genes in each cancer subtype. Figure 2A shows that AHNAK had the highest mutation frequency in most cancers, and the remaining T cell proliferation-related genes had lower mutation frequencies in most cancers. The SNV percentage analysis showed that the top 3 mutated genes were AHNAK, LIG3, and B2M, where the mutation percentages were 28%, 6%, and 5%, respectively (Figure 2B). Using the cBioPortal database (Figure 3A), we found that the types of genetic variants in T-cell proliferation-associated genes were mainly Amplification and Missense Mutation (unknown significance). To further investigate the genetic abnormalities of T-cell proliferation-related genes in cancer, we examined the percentage of SCNA. The results indicated that SCNA frequently occurred in most cancer types, with frequencies exceeding 5% of all samples (Figure 3B). As CNA plays a crucial role in regulating gene expression in tumors, we assessed the impact of CNA on gene expression. Pearson correlation analysis was conducted between gene expression and copy number using the masked copy number fragment from TCGA. The findings demonstrated significant correlations between the expression of most T cell proliferation-related genes and SCNA in various tumors (Figure 3C). For instance, the expression of citrate synthase (CS), involved in oxidative metabolism, displayed a significant association with CNA across all cancers. These results indicate that abnormalities in the copy number of T-cell proliferation-related genes are commonly observed in most cancers and can influence gene expression. We observed that T-cell proliferation-associated genes showed hypomethylation status in most cancer types (Figure 3D). The correlation analysis (Figure 3E) revealed a correlation between gene expression and DNA methylation.

### Correlation analysis of T cell proliferation-related genes with TMB and MSI

**Figure 4:**
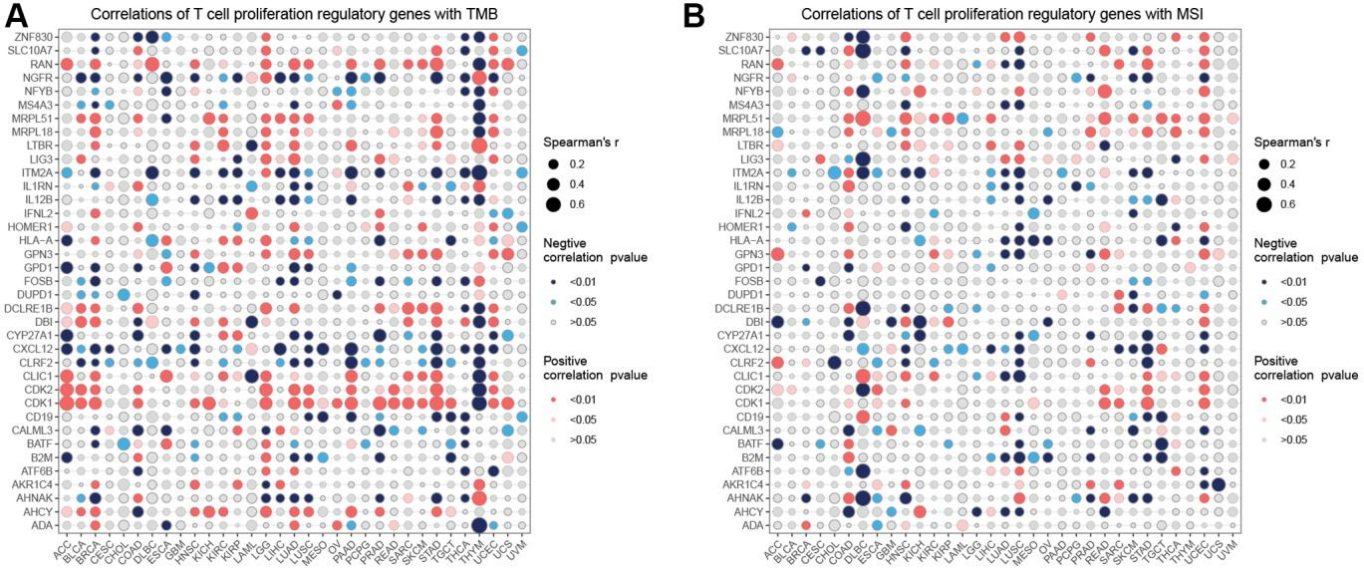
**Correlation analysis.** Correlation analysis of T cell proliferation regulatory genes with TMB (**A**) and MSI (**B**).

**Figure 5:**
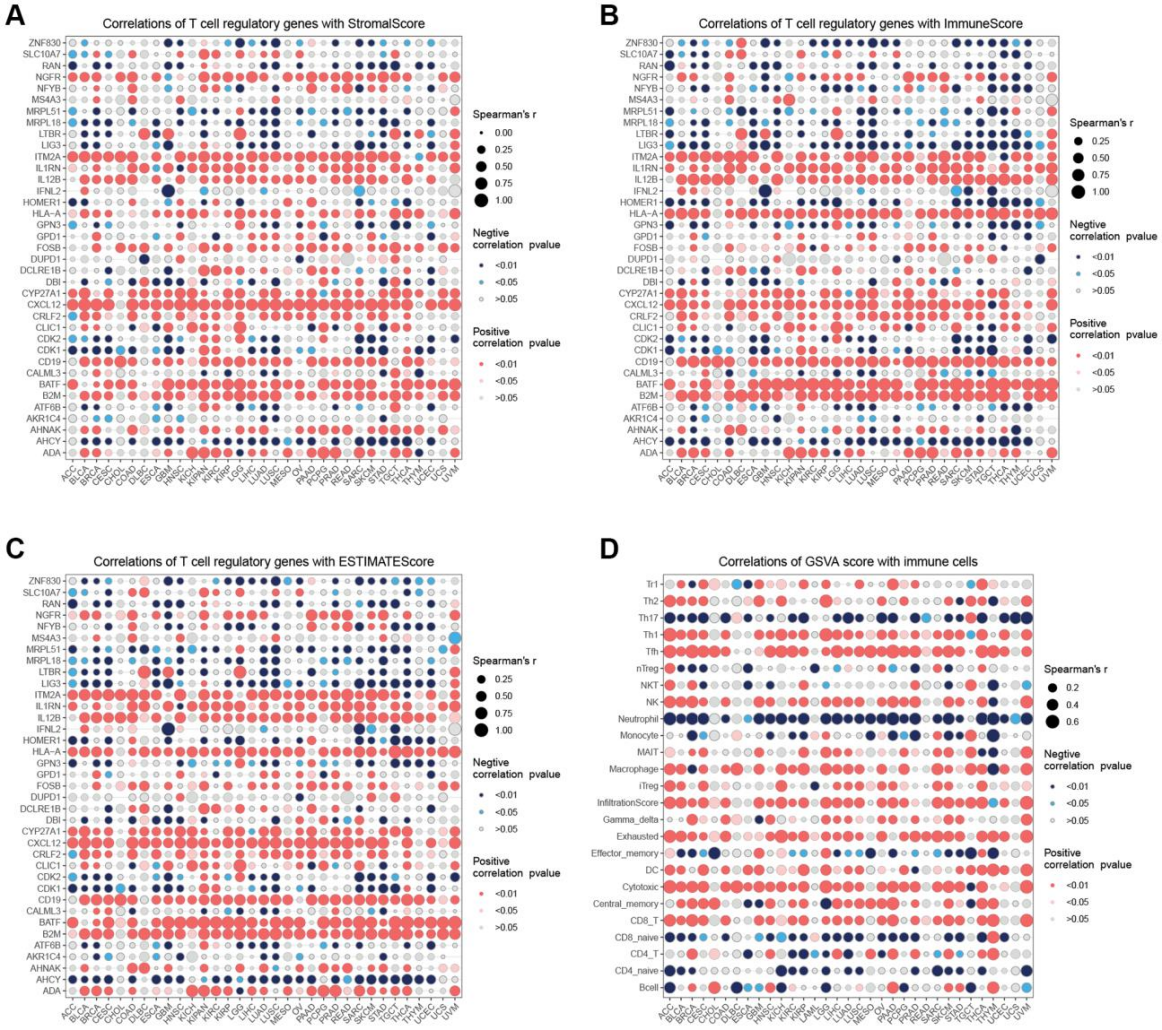
**Immune infiltration analysis.** (**A**) Correlation analysis of T cell proliferation regulatory genes with StromalScore. (**B**) Correlation analysis of T cell proliferation regulatory genes with ImmuneScore. (**C**) Correlation analysis of T cell proliferation regulatory genes with ESTIMATEScore. (**D**) Correlation analysis of T cell proliferation regulatory genes’ GSVA score with immune cells.

The correlation analysis (Figure 4A) demonstrated that CDKI exhibited a positive correlation with 21 tumors and a negative correlation with one tumor. On the other hand, CXCL12 displayed a negative correlation with 18 tumors and a positive correlation with one tumor. Regarding the correlation of T cell proliferation-related genes with MSI (Figure 4B), MRPL15 was positively correlated with 14 tumors, while negatively correlated with one tumor. The correlation between StromalScore and T cell proliferation-related genes showed (Figure 5A) that NGFR, ITM2A, IL1RN, IL12B, HLA-A, CRLF2, CXCL12, CYP27A1, CD19, B2M, BATF, and ADA were significantly positively correlated in most tumors with StromalScore; ZNF830, LIG3, LTBR, MRPL18, MRPL51, HOMER1, GPN3, CDK1, CDK2, and AHCY were significantly richly correlated with StromalScore in most tumors. The correlation analysis of ImmnueScore showed (Figure 5B) that IL12B, IL1RN, ITM2A, HLA-A, CRLF2, CXCL12, CYP27A1, CD19, B2M, BATF, and ADA were significantly positively correlated with ImmnueScore of most tumors; ZNF830, RAN, NFYB, MRPL18, LTBR, LIG3, HOMER1, GPN3, CDK2, CDK1, and AHCY were significantly negatively correlated with ImmnueScore of most tumors. In terms of ESTIMATEScore (Figure 5C), ITM2A, IL12B, IL1RN, HLA-A, CLIC1, CRLF2, CXCL12, CYP27A1, CD19, B2M, BATF, and ADA were significantly positively associated with ESTIMATEScore; ZNF830, RAN, MRPL51, MRPL18, LTBR, LIG3, HOMER1, GPN3, CDK2, CDK1, ATF6B, and AHCY were significantly negatively associated with ESTIMATEScore. The results of the correlation between T cell proliferation-related genes GSVA score and immune cells showed (Figure 5D) that Tr1, Th2, Th1, Tfh, NK, MAIT, Macrophage, Exhausted, Gamma_delta, InfiltrationScore, iTreg, DC, Cytotoxic, Central_memory, and CD8_T were significantly positively associated with GSVA scores in most tumors; Th17, Neutrophil, Effector_memory, CD8_naïve, and CD4_naïve were significantly negatively associated with GSVA scores of T cell proliferation-related genes in most tumors. These results suggest that T-cell proliferation-related genes and tumor immune microenvironment are closely related.

### Enrichment analysis

**Figure 6:**
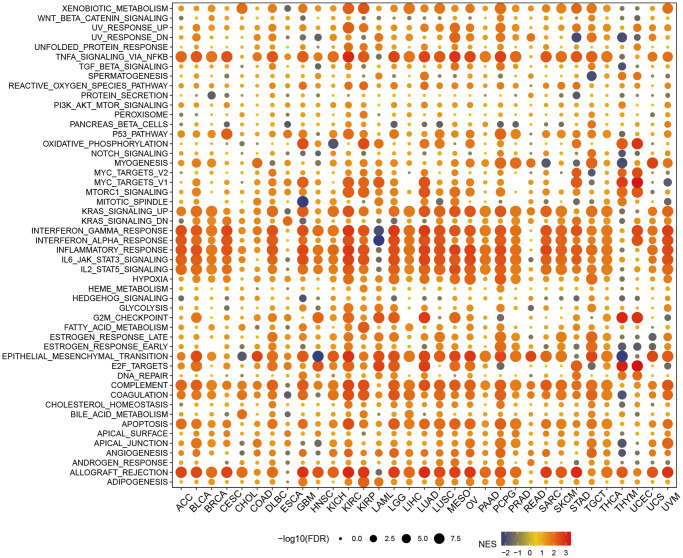
Gene set enrichment analysis in hallmark gene set.

**Figure 7:**
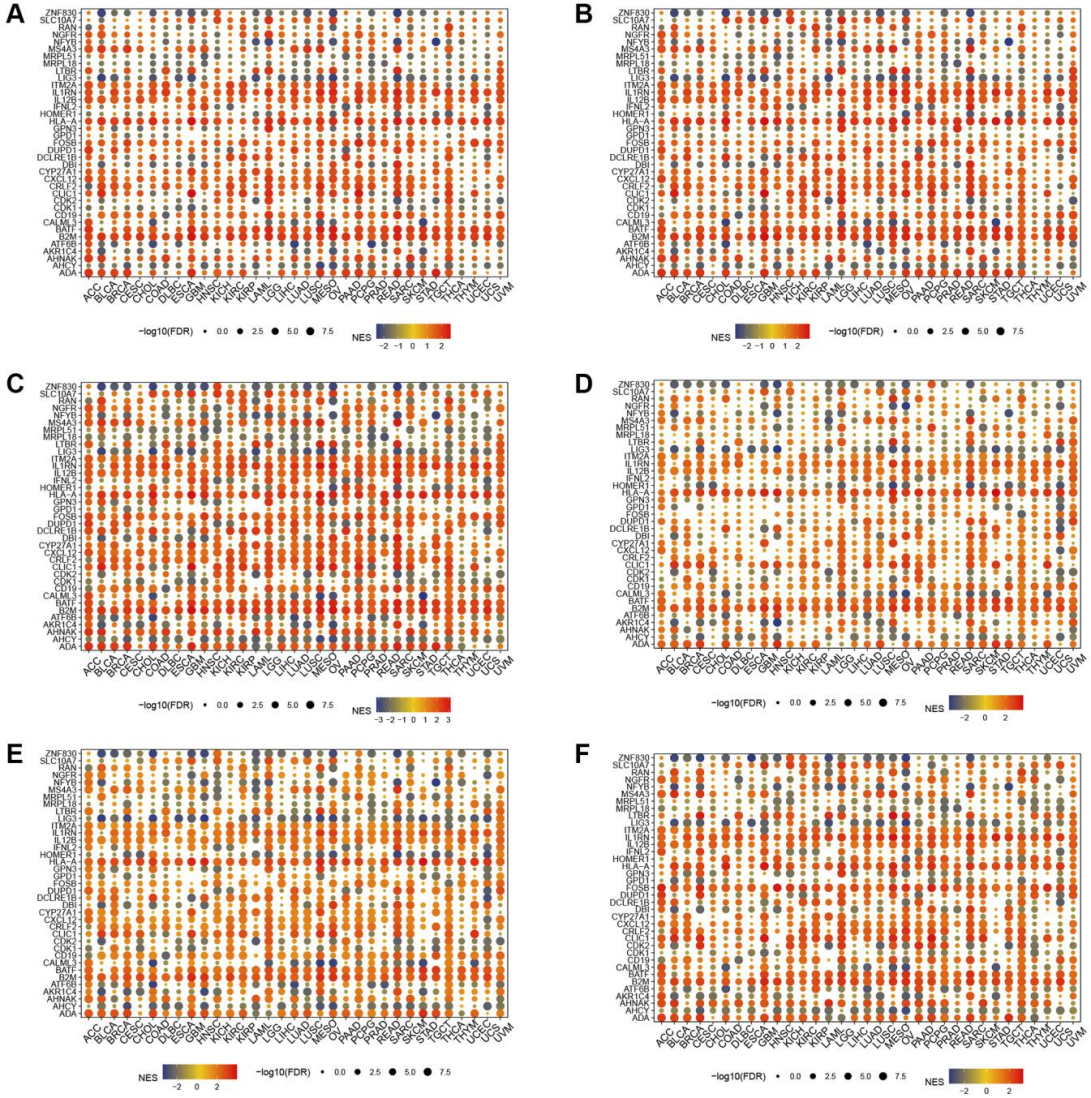
**Gene set enrichment analysis.** T cell proliferation regulatory genes in il2 stat5 signaling (**A**), il6 jak stat3 signaling (**B**), inflammatory response (**C**), interferon alpha response (**D**), interferon gamma response (**E**), and tnfa signaling via nfkb (**F**).

In the hallmark gene sets, T cell proliferation-associated genes were associated with immune-related pathways, like especially tnfa signaling via nfkb, kras signaling up, and interferon gamma response (Figure 6). We observed a robust positive association between immunological pathways and genes involved in T-cell proliferation. Therefore, we further showed the enrichment of each T cell proliferation-related gene in the immune-related pathways of each tumor, including il2 stat5 signaling (Figure 7A), il6 jak stat3 signaling (Figure 7B), inflammatory response (Figure 7C), interferon alpha response (Figure 7D), interferon gamma response (Figure 7E), and tnfa signaling via nfkb (Figure 7F).

### Activity score and drug sensitivity analysis

**Figure 8:**
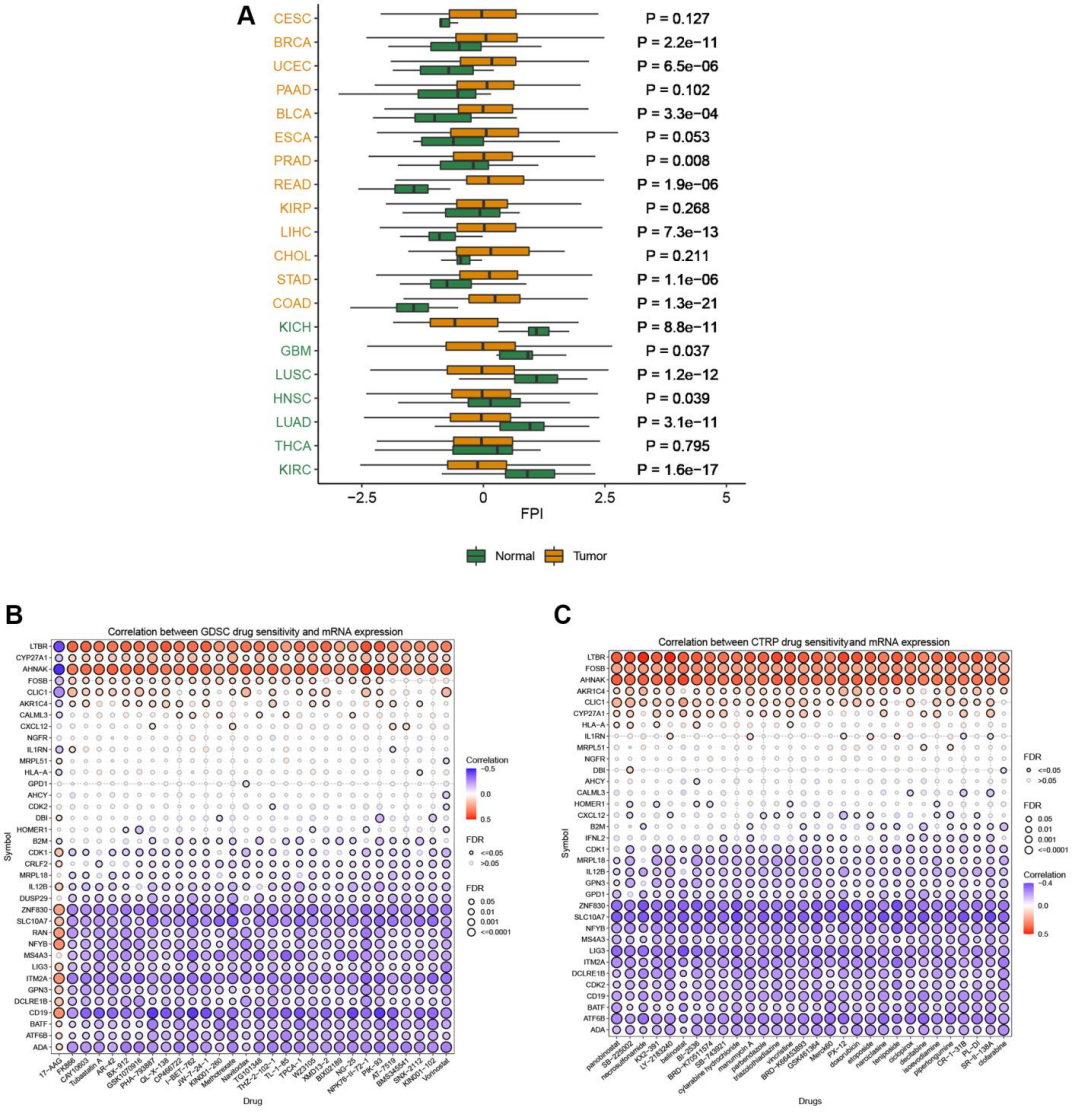
**Activity and drug analysis.** (**A**) Activity of T cell proliferation regulatory genes in different tumors. (**B**) Drug sensitivity from GDSC. (**C**) Drug sensitivity from CTRP.

T cell proliferation regulatory gene activity scores increased in CESC, BRCA, UCEC, BLCA, COAD, STAD, CHOL, LIHC, KIRP, READ, PRAD, and ESCA and decreased in KIRC, THCA, LUAD, HNSC, LUSC, GBM, and KICH (Figure 8A). Based on the results of the GDSC database (Figure 8B) and the CTRP database (Figure 8C), we identified multiple drugs that can act simultaneously on T cell proliferation regulatory genes. T-cell proliferation regulatory genes have dual effects on these drugs, such that high gene expression can lead to increased resistance or sensitivity to the drug.

### Single-cell analysis

**Figure 9:**
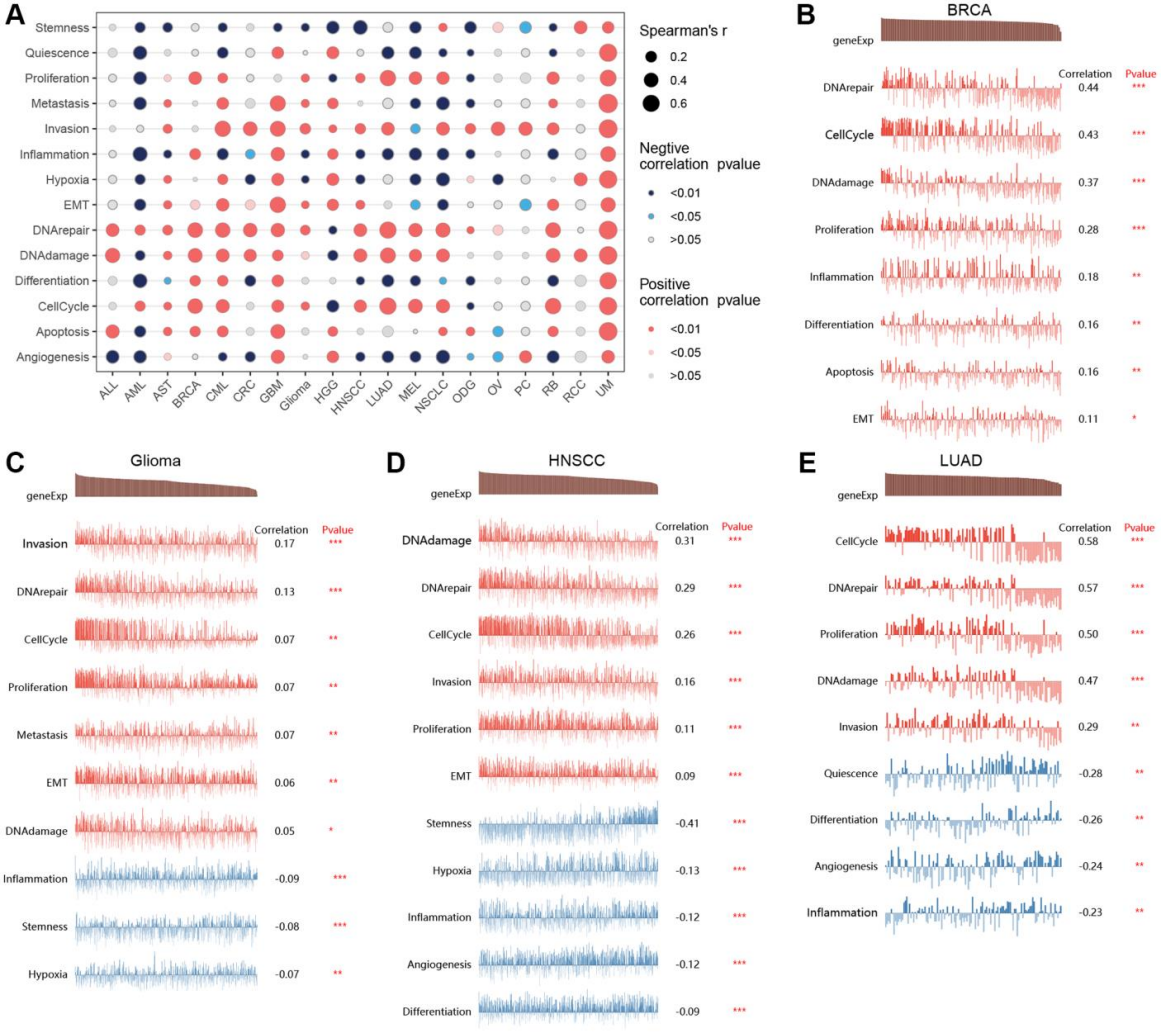
**Single-cell analysis.** The correlation between T-cell proliferation regulatory genes and cancer-related functional states (**A**). The correlation between T-cell proliferation regulatory genes and cancer-related functional states in BRCA (**B**), Glioma (**C**), HNSCC (**D**), and LUAD (**E**).

By integrating single-cell datasets, we analyzed the correlation between T-cell proliferation regulatory genes and cancer-related functional states (Figure 9A), and found that there was a correlation between T-cell proliferation regulatory genes and different cancers and functional states. Figure 9B–9E shows the correlation between T-cell proliferation regulatory genes and cancer-related functional states in BRCA, Glioma, HNSCC, and LUAD, respectively.

### Identification of 16 T-cell proliferation regulatory genes signature in pan-cancer

**Figure 10:**
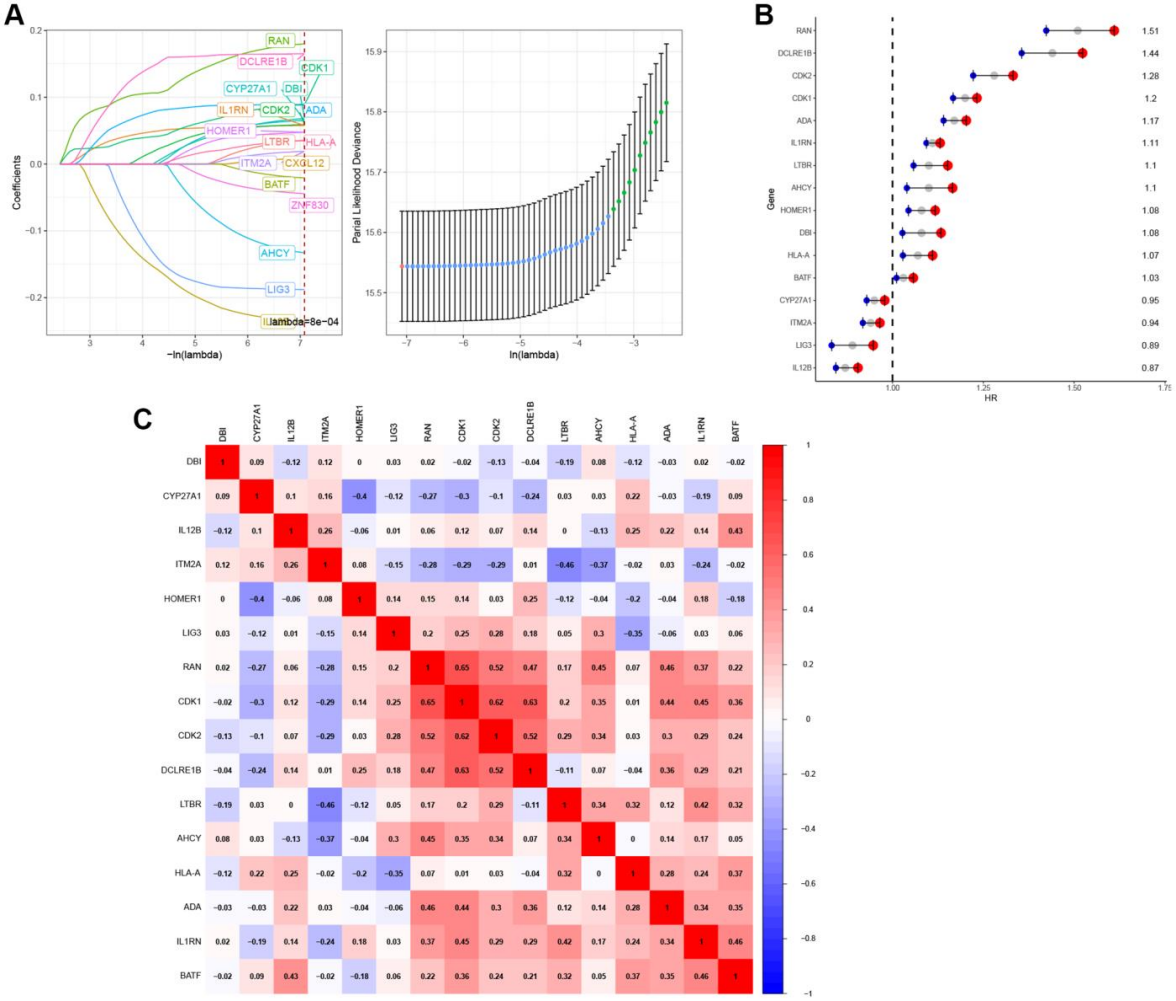
**Construction of T-cell proliferation regulatory genes-characteristic signature for pan-cancer.** (**A**) 16 T-cell proliferation regulatory genes were identified by LASSO analysis. (**B**) 16 T-cell proliferation regulatory genes were analyzed by Cox regression. (**C**) The correlation heat map of 16 T-cell proliferation regulatory genes.

In the TCGA pan-cancer training dataset, a LASSO regression analysis was conducted using 51 initial biomarkers of T cell proliferation regulatory genes (Figure 10A). This analysis identified 16 genes with non-zero correlation coefficients. To remove genes with similar expression patterns, the remaining 16 genes were selected for further model construction. Cox regression analysis was subsequently conducted on the set of 16 genes (Figure 10B). Subsequently, correlation analysis revealed that certain genes exhibited similar expression patterns (Figure 10C).

### Panorama of T-cell proliferation regulatory genes score in cancers

**Figure 11:**
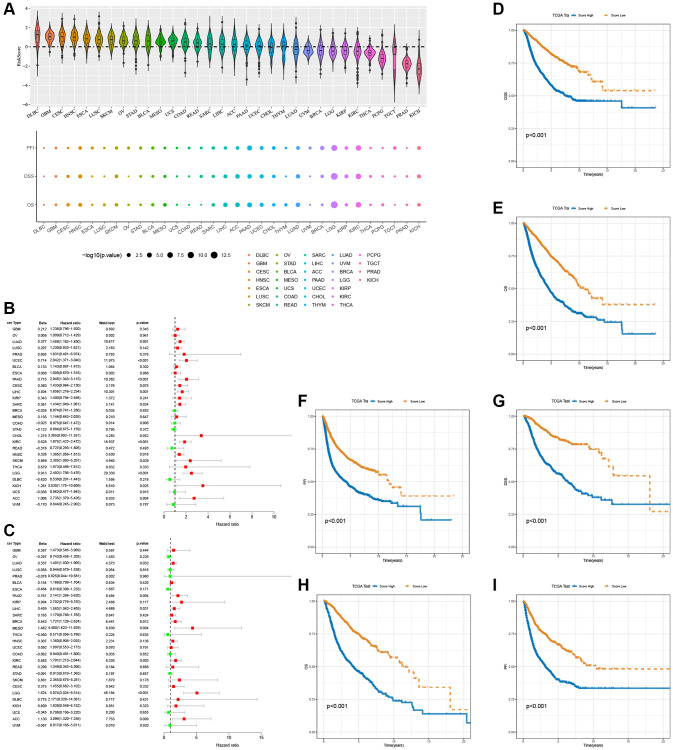
**Prognostic performance of the 16 T-cell proliferation regulatory genes score.** (**A**) Score of T-cell proliferation regulatory genes in pan-cancer. The forest map shows the effects of T cell proliferation regulatory genes score on various predictors of prognosis in the pan-cancer train cohorts (**B**) and the test cohorts (**C**), respectively. T cell proliferation regulatory genes score survival analysis in the pan-cancer train cohorts, including DSS (**D**), OS (**E**), and PFI (**F**). T cell proliferation regulatory genes score survival analysis in the pan-cancer test cohorts, including DSS (**G**), OS (**H**), and PFI (**I**).

**Figure 12:**
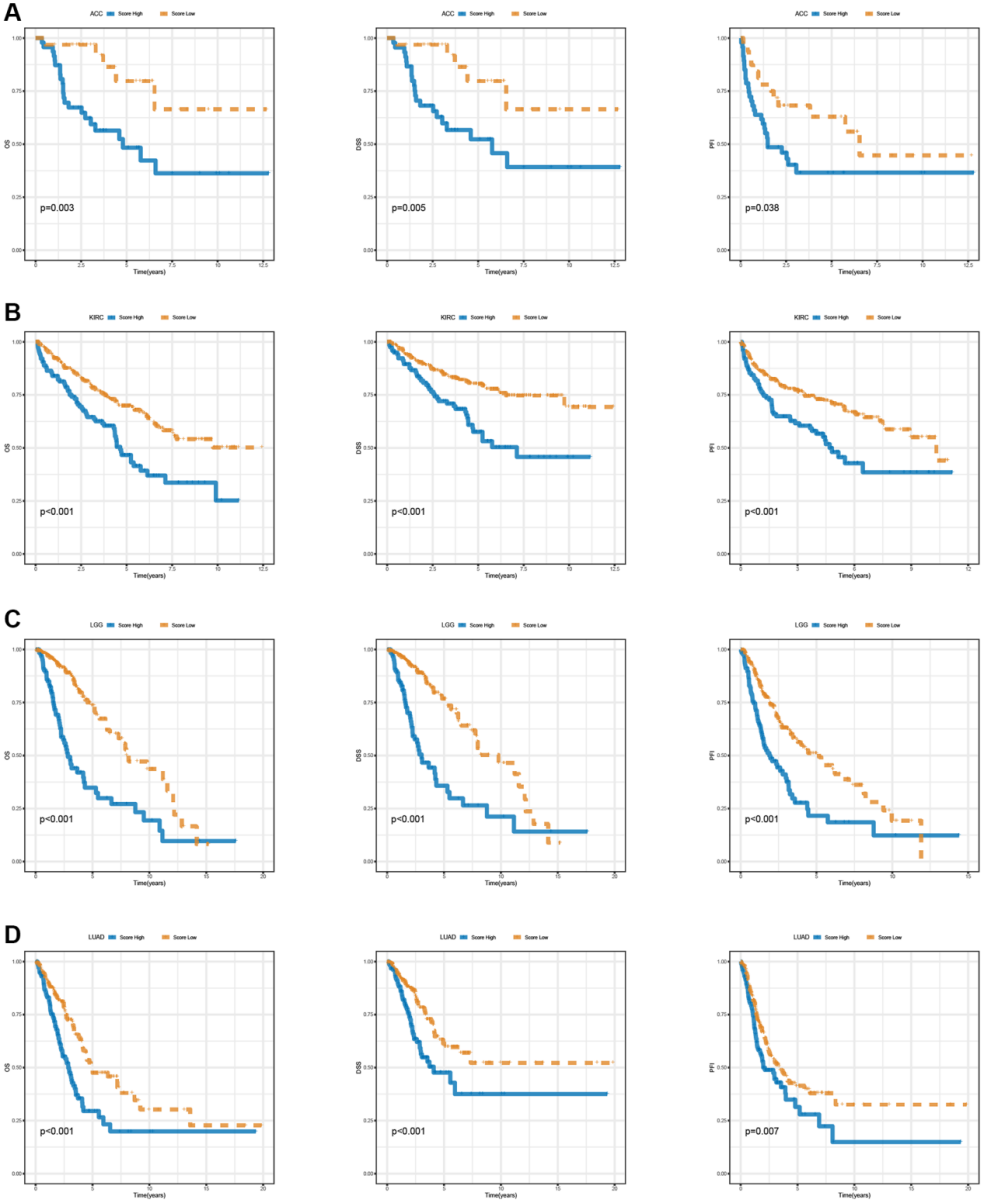
Survival analysis of T cell proliferation regulatory genes score in ACC (**A**), KIRC (**B**), LGG (**C**), and LUAD (**D**), including OS, DSS, and PFI.

In the pan-cancer context, Figure 11A illustrates the scoring of T-cell proliferation regulatory genes. Univariate Cox regression analysis was performed on both the pan-cancer training cohort (Figure 11B) and test cohort (Figure 11C) to evaluate the influence of T-cell proliferation regulatory gene scores on different predictive and prognostic outcomes. The results demonstrate a significant association between higher T-cell proliferation regulatory gene scores and adverse prognosis across most cancer types. Based on whether the T-cell proliferation regulatory gene score exceeded the population median, patients in the TCGA training cohort were classified into high-risk or low-risk groups. Compared to the low-risk group, pan-cancer patients with higher T-cell proliferation regulatory gene scores showed correlations with various unfavorable survival indicators, including DSS (Figure 11D), OS (Figure 11E), and PFI (Figure 11F). To examine the effectiveness and universality of this signature, the predictive role of T-cell proliferation regulatory gene scores was first validated in the pan-cancer test cohort. Consistent with the results obtained from the training cohort, the survival analysis of patients in the test cohort demonstrated an association between higher T-cell proliferation regulatory gene scores and poorer prognosis (Figure 11G–11I). Furthermore, it was observed that T-cell proliferation regulatory gene scores exhibited significant correlation for the prognosis of patients with ACC (Figure 12A), KIRC (Figure 12B), LGG (Figure 12C), and LUAD (Figure 12D).

### T-cell proliferation regulatory genes signature and malignant features of tumors

**Figure 13:**
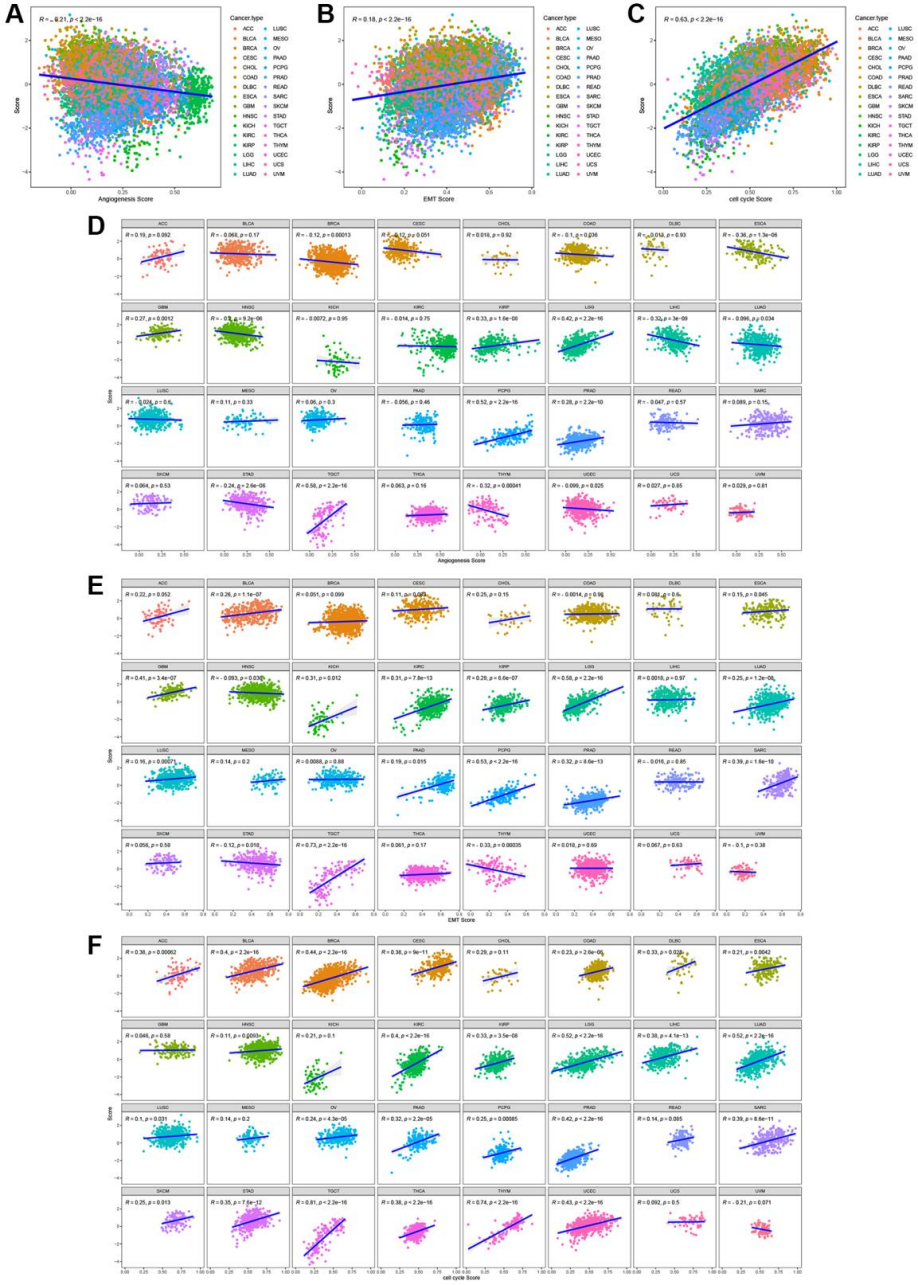
The correlation between T cell proliferation regulatory genes score and malignant features in the pan-cancer cohort (**A**–**C**) or most tumor types (**D**–**F**).

During the process of normal cell transformation into a malignant state, rapid proliferation, active epithelial-mesenchymal transition (EMT), and angiogenesis are acquired, all of which are hallmarks of cancer [23]. To examine the association between the characteristics of T-cell proliferation regulatory genes and malignant traits, we quantified the tumor’s capacities in promoting T-cell proliferation, angiogenesis, EMT, and cell cycle using the z-score algorithm. A significant correlation was observed between the z-scores of T-cell proliferation regulatory genes and both EMT and cell cycle z-scores in the entire TCGA pan-cancer cohort (Figure 13A–13C) or across most tumor types (Figure 13D–13F).

### Enrichment analysis results for high and low risk groups

**Figure 14:**
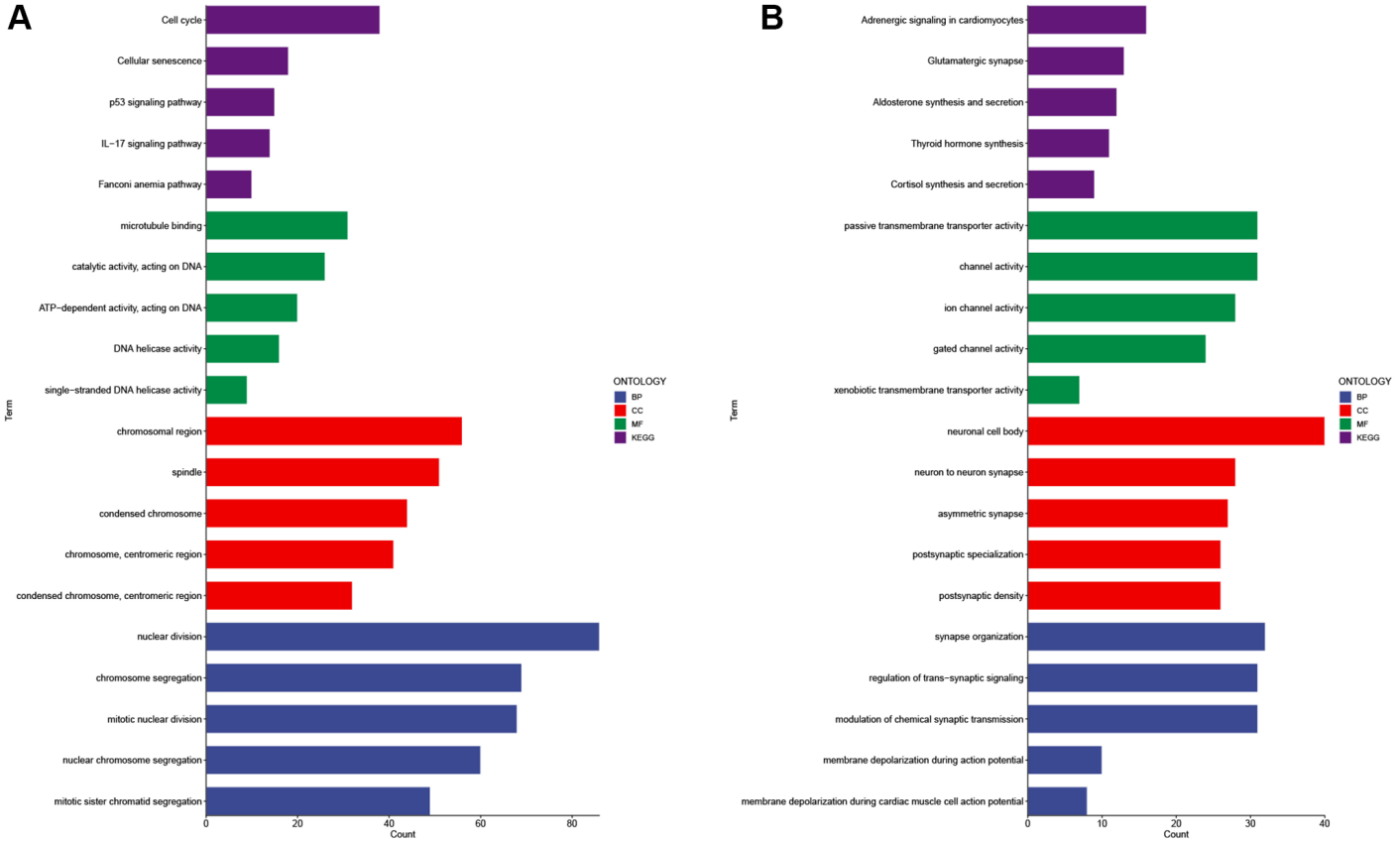
**GO and KEGG analysis of differentially expressed genes in high and low risk groups.** Enrichment analysis of up-regulated differentially expressed genes (**A**) and down-regulated differentially expressed genes (**B**). Abbreviations: GO: gene ontology; BP: biological processes; CC: cellular components; MF: molecular function; KEGG: Kyoto Encyclopedia of Genes and Genomes.

GO (Gene Ontology) and KEGG (Kyoto Encyclopedia of Genes and Genomes) analyses were conducted to explore the enriched functions of differentially expressed genes between the high-risk and low-risk groups. Up-regulated differentially expressed genes are enriched in cell cycle, cellular senescence, p53 signaling pathway, IL-17 signaling pathway, nuclear division, chromosome segregation, and mitotic karyokinesis (Figure 14A). Down-regulated differentially expressed genes were mainly enriched in Adrenergic signaling in cardiomyocytes, Glutamatergic synapse, Aldosterone synthesis and secretion, synapse organization, regulation of trans-synaptic signaling, modulation of chemical synaptic transmission (Figure 14B).

### Experimental verification

**Figure 15:**
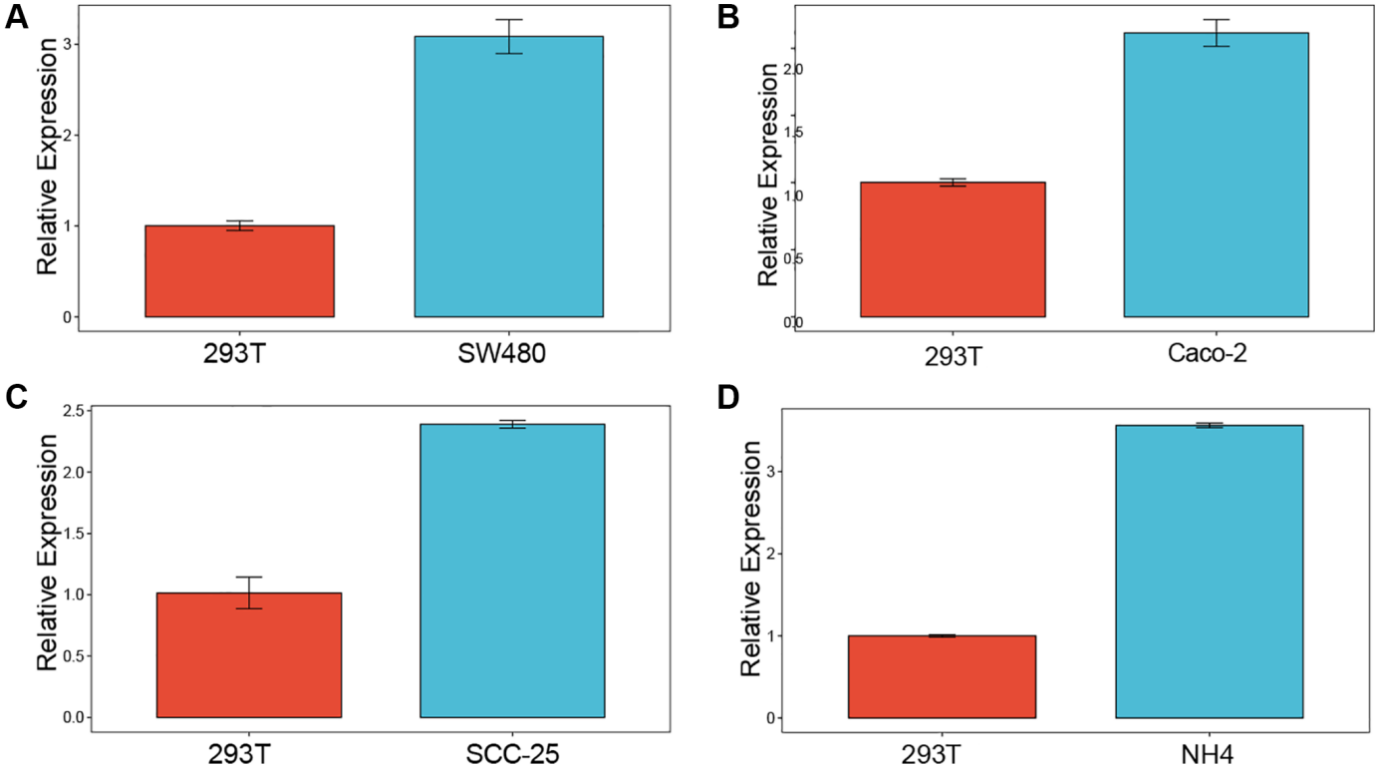
**Experimental validation.** Enrichment analysis of up-regulated differentially expressed genes (**A**–**D**) qPCR results revealed elevated levels of LTBR expression in cancer cell lines.

We investigated the expression of LTBR in normal cell line (293T) and cancer cell lines (SW480, Caco-2, SCC25, NH4) by employing qPCR. The results of the study showed that LTBR was expressed at a higher level in cancer cell lines compared to normal cell line (Figure 15A–15D).

## DISCUSSION

The development of target genes and drugs that act on T cells is an effective way to treat a variety of cancers. Understanding the role of T-cell proliferation regulatory genes in cancer is crucial for comprehending tumorigenesis and identifying potential targets for clinical therapy. Through comprehensive analysis of multiple datasets, we have conducted a systematic investigation into T-cell regulators. Our findings not only shed light on diverse potential mechanisms involving T-cell regulatory genes in cancer but also identify their associations with cancer pathways, providing preliminary insights into the overall landscape of T-cell proliferation regulatory genes in cancer.

Genes do not function in isolation and can cooperate in the context of cancer thereby mediating tumorigenesis and progression. Therefore, we investigated the common features of gene alteration and expression correlations among T cell proliferation regulatory genes. 37 T-cell regulatory genes, comprising both positive and negative regulatory genes for T-cell proliferation, were found in this study. PPI and functional similarity analysis indicated that these genes have some commonality in interconnectivity and function. In addition, some genes are located at the same chromosomal loci. Most T-cell proliferation regulatory genes are aberrantly expressed in different tumor types, while frequent CNA and differential DNA methylation play an important role. The results of our genetic investigation showed that T-cell regulatory genes had copy number variations often. Expression analysis of T-cell proliferation regulatory genes confirmed that copy number changes were positively correlated with expression, suggesting that copy number changes may affect the expression of T-cell proliferation regulators at times, which in turn promotes tumorigenesis. Epigenetic analysis has shown that aberrant methylation of genes mediates their altered expression and positively correlates methylation levels with expression. Epigenetic analysis has shown that aberrant methylation of genes mediates their altered expression and positively correlates methylation levels with expression. Hence, we propose the hypothesis that genetic and epigenetic alterations in T-cell proliferation regulatory genes may induce T-cell dysfunction and contribute to tumorigenesis under specific conditions. Several known biomarkers, including patient age, tumor type, and TMB, are positively correlated with immune checkpoint blockade (ICB) response. Among them, TMB is the most well-established marker for predicting ICB response [24]. We also investigated the correlation of T cell proliferation regulatory genes with ESTIMATEScore, ImmuneScore, and StromalScore and found that they were significantly associated with scores in a variety of cancers. This finding suggests their close association with the TIM. We quantified infiltrating immune cells that may cooperate with T cell proliferation regulatory genes in the context of TIM and found that GSVA scores of T cell proliferation regulatory genes were significantly and positively correlated with CD8 T, CD4 T, Th1, Th2, and Tfh. This finding proves the validity of the score, and also suggests that these genes mediate tumor development through T cells.

We used GSEA to find that T cell proliferation regulatory genes in the context of the hallmark gene set are associated with multiple cancer pathways, especially immune-related pathways. The interactions that occur between T cells and tumor necrosis factor (TNF)-TNF receptors expressed by other immune and non-immune cell types are essential for T cell function [25]. Tumor necrosis factor must be activated by CD40-CD40L in order to stimulate dendritic cells that produce nitric oxide synthase during T cell treatment [26]. CD4+ T cells alter tumor metabolism leading to enhanced TNF-α-dependent oxidative stress and tumor cell death [27]. One powerful T-cell effector mechanism that can eliminate antigen-negative tumor cells is TNF-mediated bystander death [28]. The primary producers of IFN-γ are T cells, NK cells, and NK T cells [29]. Regulatory T (Treg) cells promote macrophage srebp1-dependent tumor metabolic adaptations by inhibiting CD8 T cell-derived interferon-γ [30]. Galactose lectin-3 reduces chemokine production and T-cell tumor infiltration by trapping interferon-γ in the tumor stroma [31]. IFNα reprograms glucose metabolism in the HCC tumor microenvironment, thereby releasing the cytotoxic capacity of T cells and enhancing the immune response induced by PD-1 blockade [32]. PD-L1 antibodies combined with IFNα enhance tumor targeting and antigen presentation while counteracting innate or T-cell-driven upregulation of PD-L1 within the tumor [33]. NLRC5 plays a protective role for T lymphocytes against NK cell-mediated elimination during inflammatory conditions [34]. Specific targeting of CD163 TAMs (tumor-associated macrophages) mobilizes inflammatory monocytes and facilitates T cell-mediated tumor regression [35]. In the TIM, the IL-6/JAK/STAT3 signaling pathway drives tumor cell proliferation, survival, invasion, and metastasis, while concurrently suppressing anti-tumor immune responses [36]. Epidermal growth factor receptors regulate both PD-L1 expression and cell proliferation in NSCLC through the IL-6/JAK/STAT3 signaling pathway [37]. IL-2-STAT5 signaling is dependent on Mst1-Mst2 function to maintain a stable Treg cell pool and immune tolerance [38]. IL-2 signaling activates the STAT5-TPH1 pathway, which promotes 5-HTP production and subsequently triggers CD8+ T-cell depletion [39]. These findings indicate that T-cell proliferation regulatory genes interact with cancer immunity signaling pathways, potentially playing a role in suppressing tumor progression and enhancing survival across multiple cancer types.

To expand the range of drugs available for immunotherapy, we searched for targeted drugs that could act on genes regulating T cell proliferation. Panobinostat blocked the Akt/FOXM1 signaling pathway to inhibit gastric cancer cell proliferation and metastasis [40]. The combination of panobinostat and olaparib demonstrated synergistic effects, including reduced tumor burden and proliferation, increased tumor apoptosis and DNA damage, enhanced infiltration of CD8+ T cells into the tumor, and decreased expression of m2-like macrophage markers [41]. Panobinostat, an inhibitor of histone deacetylase, enhances the efficacy of chimeric antigen receptor T cells specifically in pancreatic cancer [42]. Furthermore, belinostat has been approved by the U.S. Food and Drug Administration for the treatment of relapsed or refractory peripheral T-cell lymphoma [43]. Manumycin A treatment strongly affects bone marrow mesenchymal stem cell-mediated T-cell proliferation inhibition [44]. The combination of 5-carboxy-8-hydroxyquinoline (IOX1) and doxorubicin (DOX) effectively enhanced T-cell infiltration and activity, while reducing tumor immunosuppressive factors. This liposome combination exhibited significant growth reduction in various mouse tumors, including subcutaneous tumors, *in situ* tumors, and lung metastases, and also provided long-term immune memory against tumor rechallenge [45]. DOX has been shown to eliminate myeloid-derived suppressor cells and enhance the efficacy of breast cancer against pericyte metastasis [46]. Furthermore, during therapeutic administration in cancer patients, DOX promotes antitumor CD8 T-cell responses [47]. The combination of a DOX prodrug with an erythrocyte membrane-enveloped polymer nano-vaccine enhances the immune response by upregulating the expression of dendritic cells and cytotoxic T cells in lymph nodes. This combination also increases cytokine secretion and mitigates the immunosuppressive environment by suppressing regulatory T cell expression [48]. Dox in combination with IL-12 induces the expression of NKG2D in CD8+ T cells *in vivo*, thereby enhancing NKG2D+CD8+ T-dependent antitumor immune surveillance [49]. Additionally, doxorubicin sensitizes human tumor cells to killing by NK cells and T cells through the enhancement of TRAIL receptor signaling [50]. Adriamycin contributed significantly to the enhancement of T-cell and IFN-γ immunity and also reduced the levels of immunosuppressive tumor-associated macrophages (TAMs) in tumors [51]. In highly differentiated CD8+ T cells, upregulation of miR-24 correlated with reduced DNA damage response after etoposide treatment, making them sensitive to apoptotic cell death [52]. HOXA1 knockdown LUAD cells enhanced CD8+ T cell response and increased sensitivity to etoposide in the high-risk group [53]. Mechanistically, teniposide induces DNA damage in tumor cells and activates innate immune signaling pathways such as NF-κB and the STING-dependent type I interferon signaling pathway. These pathways contribute to the activation of dendritic cells and subsequent T-cell responses. Moreover, teniposide synergizes with anti-PD1 therapy to enhance antitumor effects in diverse mouse tumor models [54]. Decitabine inhibits cytotoxicity of γδ T cells by promoting KIR2DL2/3 expression [55]. It also enhances tumor recognition by T cells through upregulation of esophageal cancer MAGE-A3 expression [56]. Furthermore, low doses of decitabine not only confer enhanced and durable anti-tumor potential to CAR-T cells through epigenetic reprogramming but also promote anti-tumor T cell responses by promoting T cell proliferation [57, 58]. Evodiamine (Isoevodiamine) suppresses non-small cell lung cancer by promoting the elevation of CD8+ T cells and concurrently downregulating the MUC1-C/PD-L1 axis [59]. Piperlongumine, an immunosuppressant, exerts a pro-oxidant effect in human T cells, leading to a decrease in T17 and enhanced T differentiation [60]. 17-AAG liposomes remodel the immunosuppressive microenvironment, leading to substantial augmentation of tumor-infiltrating T cells, decreased hypoxia levels, and reduced expression of suppressor lymphocytes [61]. Moreover, an effective tumor-killing strategy utilizing graphene oxide loaded with SNX-2112 and folic acid for ultrafast LTPTT not only restores T-cell function but also enhances natural immunity, actively contributing to tumor eradication [62]. Therefore, we anticipate that these drugs targeting T-cell proliferation regulatory genes have the potential to be ideal approaches for cancer therapy. However, further clinical studies and experimental research are needed to elucidate the potential mechanisms of action of these drugs on T-cell proliferation-regulated gene expression and their impact on cancer development.

We developed high and low risk groups and prognostic models based on genes regulating T cell proliferation. Most tumor progression occurs in high-risk patients, while low-risk patients have longer survival. We also found that scores were significantly associated with multiple malignant biological processes, including angiogenesis, epithelial to mesenchymal transition, and cell cycle, and enrichment analysis revealed relevant functions and pathways between the high- and low-risk groups, and also found that up-regulated differentially expressed genes were significantly enriched in the cell cycle. This study reveals a constitutive T-cell proliferation-regulated gene score-related feature, which contributes to the advancement of tumor research. However, the model requires further clinical validation and experimental exploration.

In conclusion, our study revealed expression and genetic alterations of T-cell proliferation regulatory genes in individual tumors. These genes are closely associated with the immune microenvironment and T cells. They participate in the activation of immune pathways in cancer. Targeting these T-cell proliferation regulatory genes may be an important approach to carrying out immunotherapy on cancer patients.

## MATERIALS AND METHODS

### Gene acquisition and related information

In this study, 37 genes were defined as T-cell proliferation regulatory genes, including interferon lambda 2 (IFNL2), lymphotoxin beta receptor (LTBR), interleukin 1 receptor antagonist (IL1RN), C-X-C motif chemokine ligand 12 (CXCL12), cytokine receptor like factor 2 (CRLF2), interleukin 12B (IL12B), nuclear transcription factor Y subunit beta (NFYB), basic leucine zipper ATF-like transcription factor (BATF), FosB proto-oncogene, AP-1 transcription factor subunit (FOSB), activating transcription factor 6 beta (ATF6B), AHNAK nucleoprotein (AHNAK), solute carrier family 10 member 7 (SLC10A7), calmodulin like 3 (CALML3), chloride intracellular channel 1 (CLIC1), RAN, member RAS oncogene family (RAN), cyclin dependent kinase 2 (CDK2), membrane spanning 4-domains A3 (MS4A3), cyclin dependent kinase 1 (CDK1), diazepam binding inhibitor, Acyl-CoA binding protein (DBI), cytochrome P450 family 27 subfamily A member 1 (CYP27A1), Aldo-Keto reductase family 1 member C4 (AKR1C4), DUPD1, glycerol-3-phosphate dehydrogenase 1 (GPD1), GPN-Loop GTPase 3 (GPN3), adenosyl homocysteinase (AHCY), adenosine deaminase (ADA), DNA ligase 3 (LIG3), integral membrane protein 2A (ITM2A), homer scaffold protein 1 (HOMER1), mitochondrial ribosomal protein L18 (MRPL18), mitochondrial ribosomal protein L51 (MRPL51), zinc finger protein 830 (ZNF830), DNA cross-link repair 1B (DCLRE1B), beta-2-microglobulin (B2M), major histocompatibility complex, class I, A (HLA-A), CD19 molecule (CD19), and nerve growth factor receptor (NGFR) [63].

### Expression and survival analysis

The pan-cancer expression matrix and survival data were obtained from the Xena database (https://xena.ucsc.edu). From tumors with available tumor and normal samples, we extracted the expression levels of 37 T-cell proliferation regulatory genes. Differential expression analysis on these genes was conducted using the Limma package [64]. We further demonstrated the expression of LTBR, the most powerful functional gene among 37 T cell proliferation-related genes, in each tumor. Differentially expressed genes (DEGs) were determined based on a threshold of FoldChange >1 and an adjusted *P*-value (FDR) less than 0.05. The impact of T-cell proliferation regulatory genes on patient prognosis was investigated using Cox analysis.

### Genetic analysis

Single nucleotide variation (SNV) data from the TCGA database were collected for 33 cancers (*n* = 8,663). The frequency (percentage) of SNV mutations in the coding region of each gene was calculated by determining the number of mutated samples out of the total tumor samples. SNV waterfall plots were generated using the mafTools R package. Genetic variant analysis of T-cell proliferation-associated genes was performed using the cBioPortal database (http://www.cbioportal.org). Copy number variation (CNV) data were obtained from the Xena database, allowing for the analysis of somatic copy number variation in T-cell proliferation-related genes across different cancer types. Bar graphs were created to visualize these findings. The correlation between somatic cell copy number and gene expression of T-cell proliferation-associated genes was simultaneously calculated and displayed using point plots. The methylation status of T-cell proliferation-related genes in tumor and normal tissues was analyzed. The correlation between the expression of T-cell proliferation-related genes and promoter methylation was also evaluated. The R package “IlluminaHumanMethylation-450kanno.ilmn12.hg19” from BioConductor was utilized to annotate the methylation probes for each gene promoter. Wilcoxon signed rank order test was employed to identify significantly hypo- or hypermethylated genes by comparing methylation levels in tumor and normal tissues, with a *P*-value cutoff of 0.05. Pearson’s correlation was calculated to assess the relationship between transcript expression of T-cell proliferation-related genes and the Beta value of promoter DNA methylation. A correlation was deemed significant if the *P*-value was < 0.05.

### TMB, MSI and immuno-infiltration analysis

The correlation between T-cell proliferation-related gene expression and tumor mutational burden (TMB) or microsatellite instability (MSI) across various tumors in the TCGA dataset was assessed using the Spearman test. The results were visualized using the “ggplot2” R package [65]. Additionally, the ESTIMATEScore, ImmuneScore, and StromalScore were computed for each tumor using the ESTIMATE algorithm, and the correlation coefficients between T-cell proliferation-related genes and these three scores were calculated using the Spearman algorithm. Gene Set Cancer Analysis can be used for immune infiltration analysis of genes [66]. The Gene Set Variance Analysis (GSVA) score, which reflects the overall genomic expression level, is positively correlated with genome-wide gene expression. Hence, a higher GSVA score in the tumor group compared to neighboring groups indicates elevated overall genome expression within the tumor group. The GSVA scores were calculated using the R package GSVA. ImmunecellAI was employed to assess immune cell infiltration. The association between immune cell infiltration and genomic expression levels was evaluated using Spearman correlation analysis, represented by a correlation coefficient.

### Enrichment analysis

To further explore the pathways by which T cell proliferation-related genes affect tumors, we calculated the fraction of T cell proliferation-related genes using gene set variation analysis at the pan-cancer level. Subsequently, based on the median scores, samples from each tumor type were categorized into two groups. Gene set enrichment analysis (GSEA) was then conducted to examine the enrichment of T-cell proliferation-related genes in immune-related pathways.

### Analysis of T cell proliferation regulatory gene activity and drug sensitivity

To examine the activity changes of T-cell proliferation regulatory genes in different tumors, we utilized ssGSEA to calculate the enrichment score (ES). By subtracting the ES of T-cell proliferation negative regulatory genes from the ES of T-cell proliferation positive regulatory genes, we obtained an activity score. The Gene Set Cancer Analysis incorporates drug and gene expression information from two databases: GDSC (https://www.cancerrxgene.org) and CTRP (https://portals.broadinstitute.org/ctrp/). GDSC includes IC50 values for 265 small molecules across 860 cell lines, along with corresponding mRNA gene expression data. CTRP compiles IC50 values for 481 small molecule drugs across 1001 cell lines, also accompanied by mRNA gene expression information. Pearson correlation analysis was performed to assess the correlations between gene mRNA expression and drug IC50 values.

### Single-cell analysis

The CancerSEA database (http://biocc.hrbmu.edu.cn/CancerSEA/home.jsp) was utilized to explore the correlation between T-cell proliferation regulatory genes and 14 cancer-related functional states. CancerSEA is a comprehensive website that allows for the investigation of various functional states of cancer cells at the single-cell level. It encompasses 14 cellular functional states, such as angiogenesis, apoptosis, cell cycle, differentiation, DNA damage, DNA repair, EMT, hypoxia, inflammation, invasion, metastasis, proliferation, quiescence, and stemness. By treating T cell proliferation-regulated genes as a gene set, we performed single-cell analyses using site default parameters.

### Model construction and prognostic analysis

We randomly assign 70% of the samples as the training set and the remaining 30% as the test set. Pan-cancer samples were divided into training and test cohorts, with T-cell proliferation regulatory genes serving as the initial biomarkers for feature training. We set the regularization of LASSO regression as a one-time SE for the most concise model. The LASSO algorithm was employed to identify prognosis-related genes. Then, we analyze their correlations at the pan-cancer level. Using a Cox proportional hazards regression model, the impact of T-cell proliferation regulatory genes on prognosis was assessed. Based on the median score of these genes, patients were categorized into high-risk and low-risk groups. The effects of these risk groups on disease-specific survival (DSS), overall survival (OS), and progression-free interval (PFI) were subsequently evaluated. The effectiveness and universality of the signature were validated in the test cohort.

### Analysis of correlation between T-cell proliferation regulatory genes score and malignant characteristics

To directly examine the association between the T-cell proliferation regulatory gene score and malignant features, we utilized the z-score algorithm through GSVA to quantify the tumor’s capabilities in promoting T-cell proliferation, angiogenesis, EMT, and cell cycle. Additionally, we analyzed the correlations between T-cell proliferation regulatory gene score and these features.

### Enrichment analysis of high and low risk groups

The patients were categorized into high-risk and low-risk groups. Differential expression analysis using the limma algorithm was conducted to identify genes that exhibited differential expression between these two groups. Subsequently, the clusterProfiler R package was employed to perform functional enrichment analyses, including GO and KEGG, on the differentially expressed genes.

### Quantitative real-time PCR

To extract total RNA from cells, we employed TRIzol reagent. Subsequently, cDNA synthesis was performed using the PrimeScript RT Reagent Kit (TaKaRa, Japan). Quantitative PCR analysis was carried out on the Roche LightCycler 480 II Real-Time PCR system (Roche, Switzerland) with FastStart Universal SYBR Green Master Mix (ROX). Gene expression levels were assessed in three replicates. The qPCR experiments utilized the following primers: Human LTBR: forward, 5′-GAAGGGTAACAACCACTGC-3′; reverse, 5′-CTTGGTTCTCACACCTGGT-3′. Human GAPDH: forward, 5′-TCAAGATCATCAGCAATGCC-3′; reverse, 5′-CGATACCAAAGTTGTCATGGA-3′. The relative gene expression levels were determined using the 2^ΔΔCt^ method. All experiments were performed in triplicate.
